# Engineering PET surface colonization with a synthetic bacterial consortium

**DOI:** 10.3389/fbioe.2026.1951515

**Published:** 2026-09-30

**Authors:** Bahareh Arab, Joseph Wortman, Boxin Zhao, C. Perry Chou, Yilan Liu

**Affiliations:** Department of Chemical Engineering, University of Waterloo, Waterloo, ON, Canada

**Keywords:** biofilm formation, enzyme secretion, PETase, polyethylene terephthalate (PET), surface roughness, synthetic bacterial consortium

## Abstract

**Introduction:**

Polyethylene terephthalate (PET) is a widely used synthetic polymer that persists in the environment because of its resistance to biological degradation. Although engineered PET hydrolases have shown improved catalytic performance, translating these advances into effective whole-cell degradation remains challenging because cells and enzymes must interact with an insoluble polymer surface.

**Methods:**

We developed a synthetic bacterial consortium to investigate early PET surface colonization and biofilm formation. The system combined Escherichia coli Nissle 1917 engineered for PETase expression with a biofilm-forming Pseudomonas putida strain evolved for growth on ethylene glycol. PET film incubation experiments, microscopy, biofilm quantification, and surface characterization were used to evaluate how extracellular enzyme targeting, consortium composition, and PET surface roughness influence microbial attachment.

**Results:**

Co-culture experiments with PET films showed that surface attachment was strongly influenced by extracellular enzyme targeting and consortium composition. The strongest and most persistent attachment was observed when extracellular enzyme availability was paired with the biofilm-forming partner. Quantitative analysis showed an approximately 1.5-fold increase in biofilm formation in the engineered consortium relative to the control. In addition, mechanical roughening of PET surfaces further increased biofilm formation, indicating that surface accessibility is an important determinant of microbial attachment.

**Discussion:**

Our findings show that PETase secretion, consortium composition, and polymer surface roughness may collectively influence biofilm formation on PET and could therefore be considered when designing future whole-cell PET bioprocessing systems.

## 1 Introduction

Polyethylene terephthalate (PET) is one of the most extensively used synthetic polymers worldwide and is produced at very large scale as a major material for food packaging and textile applications (Nisticò, 2020). Its widespread adoption is largely due to its high mechanical strength, chemical stability, transparency, and good barrier properties, which make it particularly attractive for bottles, films, fibers, and other consumer products (Dhaka et al., 2022; Ma et al., 2018). However, these same properties also make PET resistant to natural degradation, allowing it to persist in landfills and in terrestrial and aquatic environments (Han et al., 2025). In 2020, only approximately 23% of PET waste was recovered through recycling into PET materials, while the remainder was primarily disposed of through landfilling and incineration (Duan et al., 2024) (Figure 1A). Consequently, the continued accumulation of PET waste has become an important environmental concern and has increased interest in developing more sustainable recycling and degradation strategies (Joseph et al., 2024).

**FIGURE 1:**
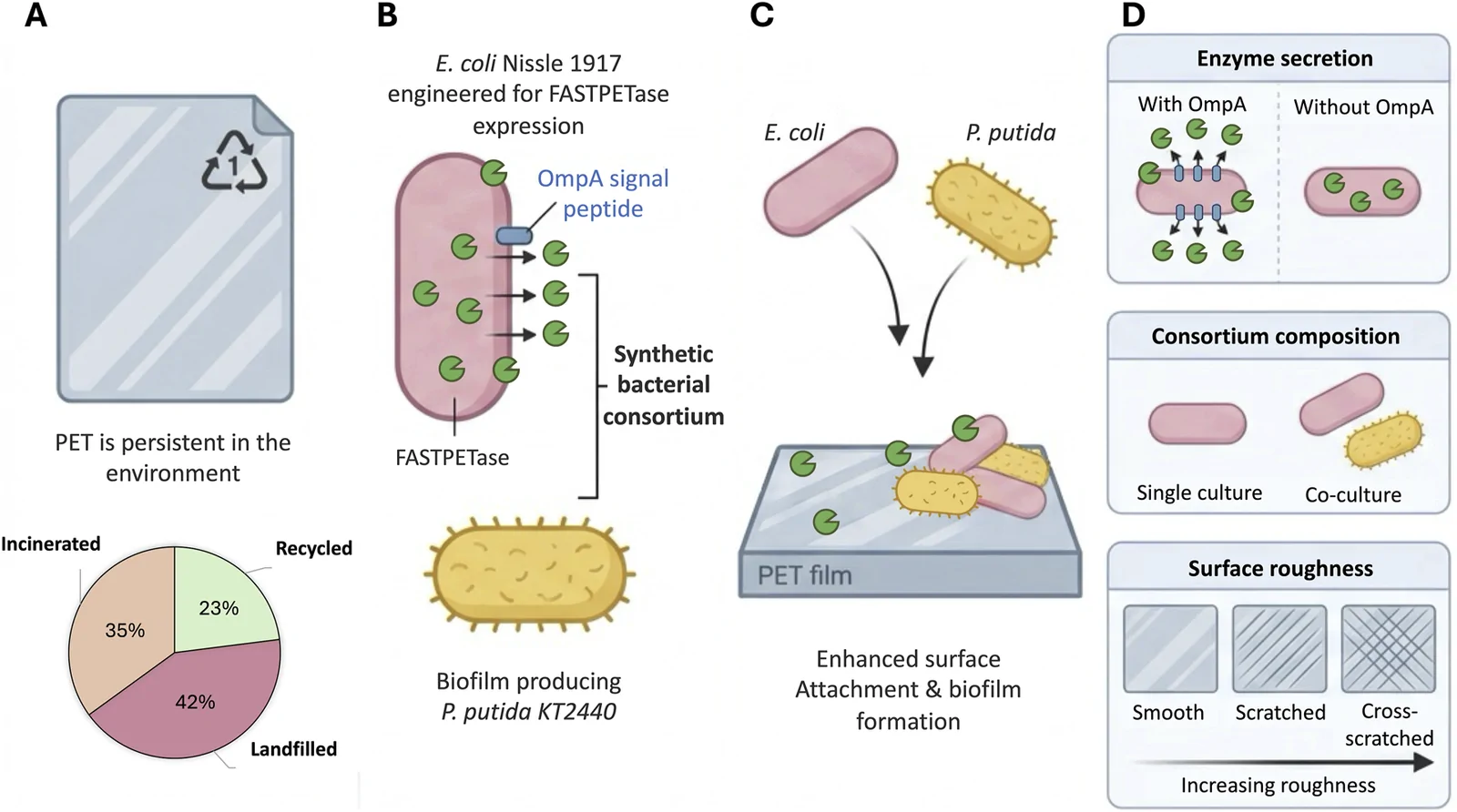
Schematic overview of the synthetic bacterial consortium strategy for enhancing PET surface attachment and biofilm formation. **(A)** PET waste persistence and end-of-life distribution. **(B)** Construction of a consortium comprising *Escherichia coli Nissle 1917* engineered for PETase expression and a biofilm-forming *Pseudomonas putida* strain, both evolved for ethylene glycol utilization. **(C)** Co-culture colonization of PET films to promote surface attachment and biofilm formation. **(D)** Experimental variables evaluated, including PETase secretion, consortium composition, and PET surface roughness. Figure created using FigureLabs.

Biological approaches to PET recycling have gained increasing attention following the discovery of the PET-degrading bacterium *Ideonella sakaiensis* and its two key enzymes, PETase and MHETase (Yoshida et al., 2016). These enzymes catalyze the hydrolysis of PET into its monomeric components, terephthalic acid (TPA) and ethylene glycol (EG) (Qiu et al., 2024), offering a potential route toward sustainable plastic recycling. Since then, substantial effort has been devoted to engineering PET hydrolases with improved catalytic activity, thermal stability, and operational robustness (Son et al., 2019; Bell et al., 2022; Lu et al., 2022). Despite this progress, efficient PET biodegradation remains challenging, particularly under process- or environmentally relevant conditions where substrate properties, enzyme stability, and mass-transfer limitations constrain overall performance (Arnal et al., 2023).

One of the primary constraints is the limited accessibility of enzymes to the polymer surface. PET is an insoluble polymer with hydrophobic surface properties and limited accessible surface area, which greatly reduces the number of ester bonds available for cleavage, restricts enzyme–substrate interactions, and makes overall degradation strongly dependent on interfacial enzyme binding and surface properties (Møller et al., 2025; Qiu et al., 2024). Recent work has shown that adsorption of PET hydrolases to the polymer surface is itself a major determinant of depolymerization efficiency and has received less attention than improvements in catalytic activity and thermostability (Wei et al., 2022). Consequently, even when enzyme variants show improved catalytic performance, overall PET degradation may remain limited when substrate accessibility and interfacial interactions are not improved.

In natural environments, microbial transformation of insoluble substrates often occurs in surface-associated communities rather than in freely suspended cells. Biofilms enable microorganisms to adhere to surfaces, establish localized high cell densities, and create a structured microenvironment that can support sustained enzyme–substrate interactions at the interface (Sonawane et al., 2022; Karygianni et al., 2020; Benes et al., 2025). This spatial organization is particularly important for the degradation of solid materials, where diffusion limitations and surface accessibility play dominant roles. Previous studies have demonstrated PET surface colonization and biofilm formation by *Rhodococcus pyridinivorans* P23 and *Pseudomonas chengduensis* BC1815, highlighting the importance of microbial attachment in PET-associated systems (Guo et al., 2023; Guo et al., 2025). In addition, surface properties such as roughness and hydrophobicity can influence microbial attachment and promote biofilm formation at the solid–liquid interface (Gao and Sun, 2021; Characklis, 1990; Van Houdt and Michiels, 2005). These features suggest that microbial attachment and biofilm formation may represent important interfacial factors for improving interaction with PET surfaces and could serve as useful complements to enzyme engineering strategies.

Synthetic microbial consortia provide a promising approach to address these challenges by distributing functional roles across different organisms. In such systems, individual species can be engineered or selected to specialize in complementary tasks, such as enzyme production, substrate utilization, and surface colonization (Lin et al., 2024). *Escherichia coli* is widely used as a host for recombinant protein expression (De Marco, 2025), while *Pseudomonas putida* is known for its metabolic versatility and strong tolerance to changing environmental and physicochemical conditions (Martinez-Garcia and de Lorenzo, 2024). Our previous work demonstrated that an evolved *Escherichia coli–P. putida* consortium can efficiently utilize PET-derived ethylene glycol as the sole carbon source (Dharmasiddhi et al., 2025). Combining these therefore offers an opportunity to couple efficient enzyme production with enhanced microbial attachment at the polymer interface.

In this study, we investigated early interfacial events that can limit whole-cell PET bioprocessing, focusing specifically on microbial attachment and biofilm formation. We developed a synthetic bacterial consortium designed to combine extracellular PETase delivery with enhanced microbial attachment to PET (Figure 1B). Specifically, we investigated how PETase secretion, consortium composition, and surface accessibility influence surface attachment and biofilm formation on PET films (Figures 1C,D). We hypothesized that effective interaction with PET depends not only on enzyme activity, but also on the ability of microbial cells to establish and maintain contact with the polymer interface. Our results show that these factors act together to shape microbial attachment to PET, highlighting interfacial processes as an important target for future studies that integrate PET-surface colonization with quantitative PET hydrolysis and monomer-utilization measurements.

## 2 Materials and methods

### 2.1 Bacterial strains and plasmids

The bacterial strains and plasmids used in this study are detailed in Supplementary Table S1. *Escherichia coli* HI-Control 10G (Lucigen, Middleton, WI, United States) was used for plasmid construction, while an evolved *E. coli Nissle* 1917 (EcNev) served as the heterologous host for PETase expression. A *P. putida* KT2440-derived strain (Ppev) from our previous work was used as the consortium partner. Both strains had been previously engineered and evolved for improved growth on ethylene glycol and Ppev showed enhanced biofilm formation (Dharmasiddhi et al., 2025; Arab et al., 2025). Three plasmid constructs were used in this study: pTrc99a-P_*J23119*_:OmpA:*FASTPETase* (OFP), encoding FASTPETase fused to the OmpA signal peptide for Sec-dependent periplasmic targeting; pTrc99a-P_*J23119*_:*FASTPETase* (FP), encoding FASTPETase without the signal peptide; and pTrc99a, which served as an empty vector control. Plasmids were introduced into EcN using standard transformation procedures. Plasmid construction was carried out using the Gibson assembly method (Gibson et al., 2009), and purified using the Qiagen Miniprep kit. Whole-plasmid sequencing was performed by Plasmidsaurus (Eugene, Oregon, United States). All primers were synthesized by Integrated DNA Technologies (IDT) (Coralville, IA, United States). The ribosome binding site (RBS) for PETase was designed using the RBS (Cetnar and Salis, 2021) Calculator in *De Novo* DNA web resources (https://salislab.net/software/). The DNA fragments were synthesized by Twist Bioscience (San Francisco, CA, United States).

### 2.2 Media and bacterial cell cultivation

All medium components were procured from Sigma-Aldrich Co. (St. Louis), except for yeast extract and tryptone, which were obtained from BD Diagnostic Systems (Franklin Lakes). *Pseudomonas putida* and *E. coli* strains were preserved as glycerol stocks at −80 °C and streaked on lysogeny broth (LB; 10 g/L tryptone, 5 g/L yeast extract, and 5 g/L NaCl) agar plates or M9 (5 g/L glucose, 6.78 g/L Na_2_HPO_4_, 3 g/L KH_2_PO_4_, 1 g/L NH_4_Cl, 0.12 g/L MgSO_4_, 0.5 mg/L thiamine, 0.011 g/L CaCl_2_, 0.5 g/L NaCl, and 1 mL/L trace elements solution containing 1.6 g/L FeCl_3_, 0.2 g/L CoCl_2_•6H_2_O, 0.1 g/L CuCl_2_, 0.2 g/L ZnCl_2_•4H_2_O, 0.2 g/L NaMoO_4_, 0.05 g/L H_3_BO_3_ in 0.1 M HCl) agar plates, followed by incubation at 30 °C for 14–16 h. When required, cultures were supplemented with ampicillin (100 mg/L). In M9EG, medium, 10 g/L of ethylene glycol (E.G.,) was added as the sole carbon source replacing glucose. Culture samples were appropriately diluted using a 0.15 M saline solution to measure cell density (OD_600_) with a spectrophotometer (GENESYS™ 40/50 Vis/UV-Vis, Thermo Fisher Scientific Inc.). For the preparation of cell-free medium, culture samples were centrifuged at 17,000 g for 1 min and then filtered through a 0.2 μm syringe to ensure sterility.

### 2.3 Enzyme purification

Thirty milliliters of Terrific Broth (TB) were inoculated with overnight cultures of E(FP) or E(OFP) to an initial OD_600_ ∼ 0.1. E(FP) served as the matched control lacking the OmpA signal peptide; otherwise, the strains were cultivated and processed in parallel under identical conditions. Cultures were incubated at 30 °C with shaking at 280 rpm for 16 h. Cells were removed by centrifugation (10,000 × g, 4 °C, 10 min), and the supernatant was collected and filtered to obtain cell-free medium (CFM). A portion of the CFM was used directly for SDS–PAGE analysis, while the remaining supernatant was subjected to His-tag affinity purification according to the manufacturer’s instructions. Briefly, the clarified CFM was loaded onto a 1 mL His-tag affinity column pre-equilibrated with binding buffer. The column was washed with a low concentration of imidazole to remove non-specifically bound proteins, and FASTPETase was eluted using an imidazole-containing buffer. Protein concentration in the purified extracellular fractions was estimated using the Bradford assay with bovine serum albumin as the standard. The cell pellet was subjected to osmotic shock using sucrose–Tris–EDTA buffer followed by incubation in ice-cold water to collect the periplasmic fraction. The remaining pellet was lysed to obtain cellular fraction.

### 2.4 SDS-PAGE analysis

For SDS–PAGE, 25 μL of each sample was mixed with 5 μL of 6× loading buffer, heated at 95 °C for 5 min, and 10 μL was loaded onto the gel. Protein bands were visualized using Coomassie Brilliant Blue R-250 staining.

### 2.5 Co-culture preparation and PET film incubation

Commercially available transparent amorphous PET film with a thickness of 0.25 mm was purchased from Goodfellow (Cambridge, UK; product no. ES30-FM-000145) and used as received. The film was cut into 1 cm × 1 cm pieces before use. For co-culture preparation, the two strains were combined at a 1:1 ratio based on equal OD_600_ contributions. The OD_600_ of each overnight seed culture was measured separately, and the required volume of each culture was calculated so that each strain contributed one-half of the target initial OD_600_. The calculated culture volumes were centrifuged separately, the supernatants were removed, and the cell pellets were resuspended in equal volumes of fresh modified M9E.G., medium before being combined. For clarity, the five experimental conditions were designated as C0, PE(C), E(OFP), PE(FP), and PE(OFP). C0 represents the cell-free medium control. PE(C) represents the control co-culture of the evolved *P. putida* strain with *E. coli Nissle* 1917 harboring the empty vector. E(OFP) denotes the *E. coli* monoculture expressing OmpA-FASTPETase, while PE(FP) and PE(OFP) denote co-cultures of the evolved *P. putida* strain with *E. coli* expressing FASTPETase without and with the OmpA signal peptide, respectively. These cultures were grown overnight in modified M9E.G., medium at 30 °C with shaking at 220 rpm and used as seed cultures for subsequent experiments. Experimental cultures were then carried out in tubes by inoculating fresh M9E.G., medium with the seed cultures to an initial OD_600_ of approximately 0.2. The PET films were sterilized by immersion in 70% ethanol for 5 min, air-dried under sterile conditions, and added to each tube at the start of cultivation. Cultures were incubated at 30 °C with shaking at 220 rpm, and each condition was prepared in triplicate. For light microscopy experiments, cultures containing PET films were incubated for up to 6 days, and films were collected on days 2, 4, and 6 for imaging. For biofilm quantification, cultures containing PET films were incubated for 48 h, after which the PET films were carefully removed for biofilm quantification.

### 2.6 Biofilm quantification on PET films

Following incubation, films were removed and gently washed three times with phosphate-buffered saline (PBS) to eliminate non-adherent cells, then air-dried. Adherent biofilms were stained with 0.4% (w/v) crystal violet for 15 min, followed by washing to remove excess dye. The bound stain was solubilized using 70% ethanol, and absorbance was measured at 570 nm using a spectrophotometer (GENESYS™ 40/50 Vis/UV-Vis, Thermo Fisher Scientific Inc.). Because the cultures reached different final cell densities, crystal-violet absorbance was normalized to the corresponding culture OD_600_ (A_570_/OD_600_) to account for differences in overall culture growth.

### 2.7 Light microscopy imaging

PET film surfaces were imaged using an upright light microscope (Axiolab 5, ZEISS, Germany) under brightfield illumination. Prior to imaging, samples were washed with ethanol and air-dried to remove non-adherent cells. The films were then placed on microscope slides and imaged using a ×40 objective lens to capture overall surface coverage and bacterial attachment.

### 2.8 Scanning electron microscopy (SEM)

Following incubation, PET films were gently washed three times with PBS to remove unbound cells and loosely associated biofilm and then sprayed with 70% ethanol. The films were then air-dried without an additional chemical fixation step prior to SEM analysis. PET film samples were mounted onto aluminum stubs using conductive carbon tape and sputter-coated with a 15 nm layer of gold using a sputter coater (Polaron Instruments E5100, Quorum Technologies) under an argon atmosphere to improve surface conductivity and minimize charging. The coated samples were then imaged using a scanning electron microscope (VEGA, Tescan, Czech Republic) operated under high vacuum. Surface morphology and bacterial attachment were visualized using secondary electron imaging at an accelerating voltage of 20.0 kV, a magnification of approximately 5.00 k × , and a working distance of approximately 10.6–10.7 mm, with scan speeds of 3–4.

### 2.9 16S rRNA sequencing

Following incubation, culture samples from PE(FP) and PE(OFP) were collected for microbial community composition analysis. Genomic DNA was extracted using the DNeasy Blood and Tissue Kit (Qiagen). The V3–V4 region of the 16S rRNA gene was amplified using the forward primer 5′-CCTACGGGNGGCWGCAG-3′ and reverse primer 5′-GACTACHVGGGTATCTAATCC-3′, as described by (Klindworth et al., 2013). Amplicon sequencing was performed at the Waterloo Genomics Surveillance Centre using an Illumina NextSeq 2000 instrument with a paired-end 2 × 300-bp read configuration. Sequence data were analyzed using the Illumina DRAGEN 16S Plus App version 1.0.0, with read quality control enabled (analysis pipeline version 0.7.0; DRAGEN version 4.4.4-19). Taxonomic classification was performed using the DRAGEN k-mer classifier against the RefSeq-RDP-v1 database, comprising full-length NCBI RefSeq 16S rRNA gene sequences supplemented with sequences from the Ribosomal Database Project. Relative abundance was calculated as the proportion of classified reads assigned to each taxon.

### 2.10 Surface roughening and surface characterization of PET films

PET films were mechanically roughened using a Universal Micro-Tribometer UNMT-2MT system (Centre for Tribology Inc., CETR; now Bruker). Norton® 100C medium-grit sandpaper was mounted onto the compression attachment of a 10 kg load cell. Films were subjected to a controlled normal load of 1,000 g while the instrument stage was moved in a 20 mm forward–backward cycle at 5 mm/s for 3 min. Two roughening patterns were prepared: single-direction scratching and cross-scratching. For cross-scratched films, the abrasion procedure was repeated after rotating the film by 90°, generating perpendicular surface features. Untreated PET films were used as smooth controls. Following roughening, PET films were cleaned and prepared for downstream biofilm assays or surface characterization. Surface morphology and roughness were assessed using optical microscopy, profilometry, and micro ATR-FTIR analysis. Optical images were collected using a Dino-Lite® digital microscope. Surface roughness profiles were obtained using a KLA Tencor P-6 profilometer over an approximately 1.3 mm scan length at multiple positions across each film. Primary and roughness profile parameters were calculated according to ISO 4287 using the profilometer analysis software. Because the untreated PET control exhibited slight sample warpage, a Gaussian cut-off filter was applied to remove the form component prior to calculating roughness parameters for the control surface. Primary profile parameters were used to characterize the mechanically roughened surfaces, where the large abrasion features represent the surface topography of interest. Micro ATR-FTIR spectra were collected using a Thermo Fisher Scientific Nicolet™ iN10 FTIR microscope equipped with a germanium slide-on ATR crystal.

### 2.11 Statistical analysis

Statistical analyses were performed using Microsoft Excel. Data are presented as the mean ± standard deviation of three biological replicates (n = 3). The effects of consortium composition and PET surface treatment were evaluated using two-way ANOVA with replication. Tukey’s honestly significant difference (HSD) test was subsequently used for pairwise comparisons. Statistical significance was defined as p < 0.05.

## 3 Results

### 3.1 OmpA fusion increases extracellular detection of FASTPETase

To evaluate whether the OmpA signal peptide promotes extracellular availability of FASTPETase, *E. coli* strains expressing FASTPETase with (E(OFP)) or without (E(FP)) the signal peptide were analyzed. The OmpA-FASTPETase construct includes a C-terminal His-tag used for purification (Figure 2A). Cell-free medium (CFM) from monocultures was examined by SDS–PAGE, and extracellular proteins were further enriched by His-tag affinity purification.

**FIGURE 2:**
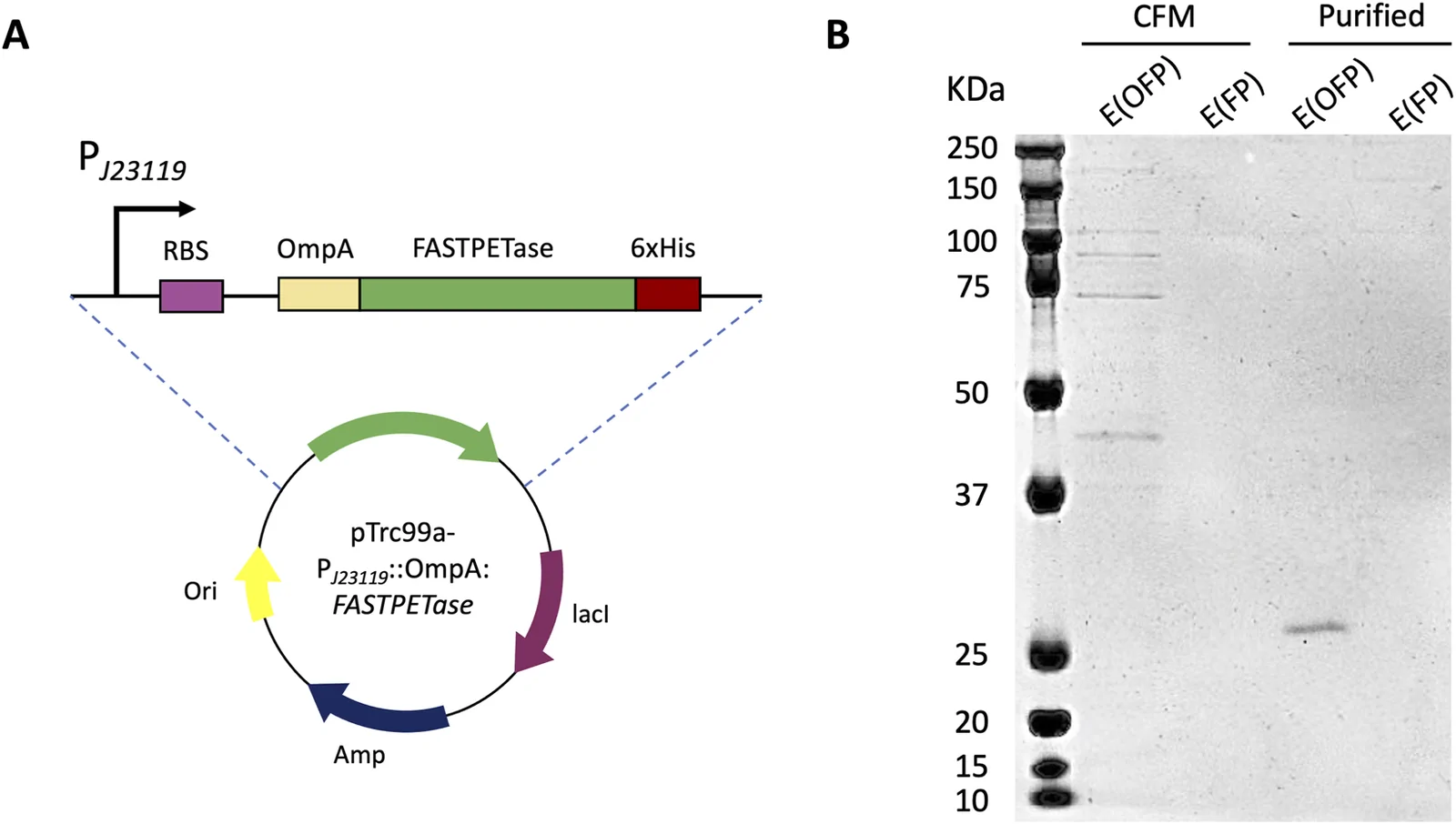
Expression and extracellular detection of FASTPETase. **(A)** Schematic diagram of the vector construction for pTrc99a-P_*J23119*_:OmpA:FASTPETase expression. **(B)** SDS–PAGE analysis of cell-free medium (CFM) and purified fractions from E(FP) and E(OFP).

SDS–PAGE analysis of the CFM showed minimal detectable protein at the expected molecular weight for both strains. However, following purification, a distinct band corresponding to FASTPETase (∼29 kDa) was observed in the E(OFP) sample, whereas no comparable band was detected for E(FP) (Figure 2B). Consistent with this result, Bradford analysis estimated the purified protein concentration from E(OFP) to be approximately 1 mg/L, while no detectable protein was recovered from the corresponding E(FP) fraction under the same conditions. Together, the SDS–PAGE and Bradford results demonstrate that FASTPETase was recovered from the extracellular fraction of E(OFP), but not from the matched E(FP) control, indicating that extracellular enzyme recovery was associated with the presence of the OmpA signal peptide under identical cultivation and purification conditions. Subcellular fractionation of E(OFP) further revealed a prominent band at ∼29 kDa in the periplasmic fraction. Although the remaining cellular lysate contained a more complex protein profile, no comparably prominent band was observed at the same apparent molecular mass (Supplementary Figure S1). This pattern supports preferential periplasmic localization of OmpA-targeted FASTPETase and provides complementary evidence for its extracellular recovery from the culture medium.

Because PET is an insoluble substrate, extracellular availability of PETase is expected to increase the opportunity for enzyme–surface contact. This result provided a basis for evaluating whether extracellular PETase availability influences PET surface colonization in the subsequent co-culture experiments.

### 3.2 Increased PET surface attachment with PETase secretion and co-culture

To examine how PETase expression and co-culture with *P. putida* influence microbial attachment to PET, PET films were incubated in M9E.G., with the five experimental conditions described in the Methods section, namely, C0, PE(C), E(OFP), PE(FP), and PE(OFP), and monitored over 6 days by light microscopy (Figure 3).

**FIGURE 3:**
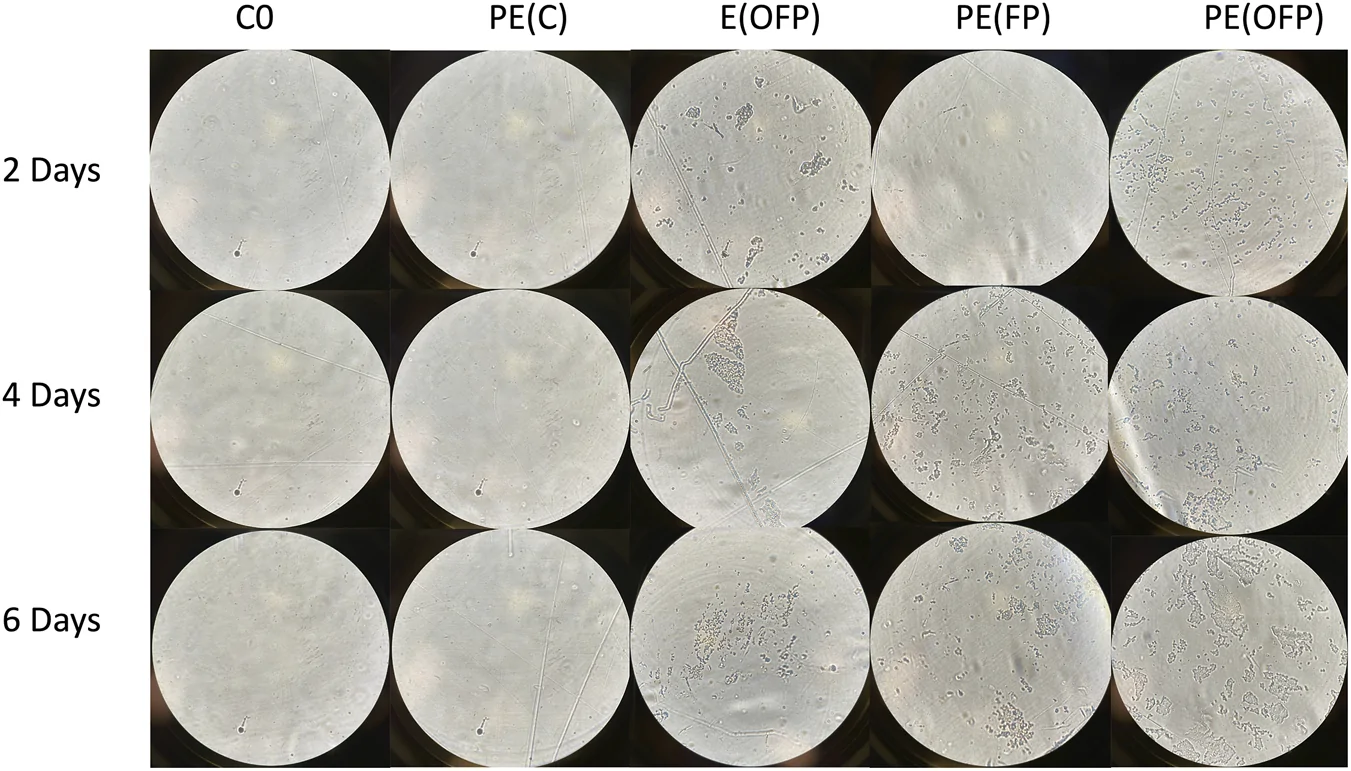
Light microscopy images of PET films incubated with different bacterial conditions over time. Representative light microscopy images of PET films incubated with C0, PE(C), E(OFP), PE(FP), and PE(OFP) for 2, 4, and 6 days. C0 represents the cell-free medium control; PE(C), the control co-culture of *Escherichia coli* Nissle 1917 carrying the empty vector with *Pseudomonas putida*; E(OFP), *E. coli* expressing OmpA-FASTPETase alone; PE(FP), the co-culture of *P. putida* with *E. coli* expressing FASTPETase without the OmpA signal peptide; and PE(OFP), the co-culture of *P. putida* with *E. coli* expressing OmpA-FASTPETase.

Clear differences in surface attachment were observed among the conditions. As expected, no cells were detected on PET films incubated in the cell-free medium control (C0), and the film surfaces remained clear throughout the experiment. Similarly, the control co-culture PE(C), consisting of *E. coli* carrying the empty vector together with *P. putida*, showed little to no visible attachment over the 6-day period, indicating that the presence of the co-culture alone was not sufficient to promote stable adhesion to the PET surface. In contrast, PETase-expressing conditions showed measurable attachment to the films. In E(OFP), which represents *E. coli* expressing OmpA-FASTPETase alone, attached cells were visible as early as day 2, suggesting that OmpA-FASTPETase expression was associated with early interaction with the polymer surface even in the absence of *P. putida*. In PE(FP), where *E. coli* expressing FASTPETase without OmpA was co-cultured with *P. putida*, attachment became more apparent by day 4 and remained detectable on day 6, suggesting that co-culture promoted PET surface association even in the absence of PETase export.

The strongest and most persistent attachment was observed in PE(OFP), the co-culture combining *P. putida* with *E. coli* expressing OmpA-FASTPETase. In this condition, substantial surface-associated cells were observed from day 2 onward and remained detectable after ethanol washing. These observations indicate that the greatest PET surface attachment occurred when extracellular PETase expression was combined with the co-culture partner.

### 3.3 SEM analysis confirms enhanced bacterial attachment in engineered consortium conditions

To further examine bacterial attachment to PET surfaces, PET films in selected conditions including PE(C), E(OFP), PE(FP), and PE(OFP) were analyzed by scanning electron microscopy (SEM) (Figure 4). Consistent with the light microscopy results, PET films from the control co-culture PE(C) showed smooth surfaces with no detectable bacterial attachment. In E(OFP), corresponding to *E. coli* expressing OmpA-FASTPETase alone, sparse bacterial cells were observed on the PET surface, indicating limited but detectable attachment. In PE(FP), where *E. coli* expressing FASTPETase without OmpA was co-cultured with *P. putida*, a higher density of cells was present, forming small aggregates on the film surface that are consistent with early-stage biofilm formation. The highest degree of surface coverage was observed in PE(OFP), the co-culture of *P. putida* with *E. coli* expressing OmpA-FASTPETase. In this condition, dense clusters of bacterial cells were distributed across the PET surface, indicating substantially stronger and more extensive attachment than in the other conditions. These observations are consistent with the light microscopy results and further support the combined contribution of PETase export and co-culture interactions to stable surface attachment.

**FIGURE 4:**
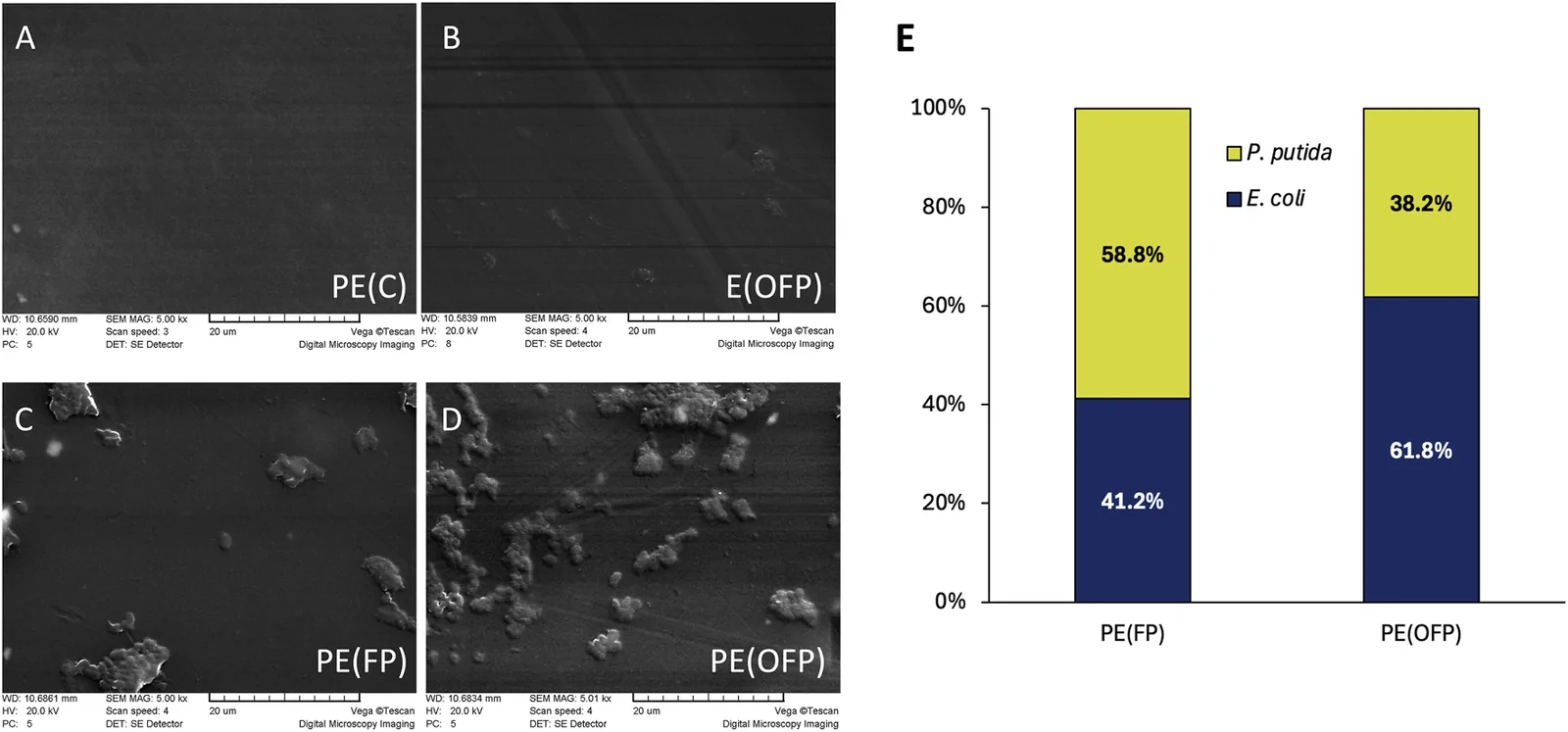
Scanning electron microscopy images of PET films incubated with selected bacterial conditions and their subpopulation composition. Representative scanning electron microscopy (SEM) images of PET films incubated with **(A)** PE(C), **(B)** E(OFP), **(C)** PE(C), and **(D)** PE(OFP). PET films were washed, sputter-coated with gold, and imaged using secondary electron detection. **(E)** Endpoint relative population composition of the PE(FP) and PE(OFP) co-cultures, determined by 16S rRNA sequencing.

Given the substantial bacterial accumulation observed in both PE(FP) and PE(OFP), the endpoint population composition of these co-cultures was further analyzed using 16S rRNA sequencing (Figure 4E). The results showed that both conditions contained mixed populations of *E. coli* and *P. putida*, with a 61.8% relative abundance of *E. coli* in PE(OFP) compared to 41.2% abundance in PE(FP). The higher relative abundance of *E. coli* in PE(OFP) indicates that the OmpA-FASTPETase condition was associated with a difference in endpoint consortium composition. This observation is consistent with the enhanced surface colonization observed in PE(OFP), although the sequencing results do not establish the mechanism underlying this difference. Despite the clear differences in bacterial accumulation, no visible surface pitting or erosion was observed by SEM within the experimental timeframe. However, SEM was used only for qualitative surface imaging and does not establish whether PET hydrolysis occurred.

### 3.4 Surface roughening modifies PET surface topography

Since PET films provide a relatively smooth interface that may limit bacterial retention, controlled mechanical roughening was introduced as a physical strategy to increase surface features available for microbial attachment. Two roughening patterns were tested: single-direction scratching, which generated linear abrasion features, and cross-scratching, which introduced a second perpendicular abrasion pattern to increase the density and distribution of surface features.

Optical microscopy showed limited visible features on smooth PET films, whereas single-direction scratching produced linear marks with clear gaps between roughened regions (Figures 5A,B). Cross-scratched films displayed denser and more uniformly distributed surface features, indicating that the second abrasion direction introduced additional features between the initial scratches, although some unmodified regions remained visible (Figure 5C). Profilometry confirmed that mechanical roughening substantially altered PET surface topography. Following Gaussian filtering to remove slight sample warpage, untreated PET showed only small roughness height deviations, with low average roughness (Ra = 0.0066 ± 0.0012 µm), root-mean-square roughness (Rq = 0.0105 ± 0.0036 µm), and peak-to-valley height (Rz/Rt = 0.129 ± 0.090 µm), with no peaks exceeding the ±0.5 µm threshold for peak-density analysis (RPc = 0 mm^−1^) (Supplementary Figure S2A; Supplementary Tables S2 and S3). In contrast, both abraded surfaces showed surface height deviations several hundred-fold greater than the untreated control.

**FIGURE 5:**
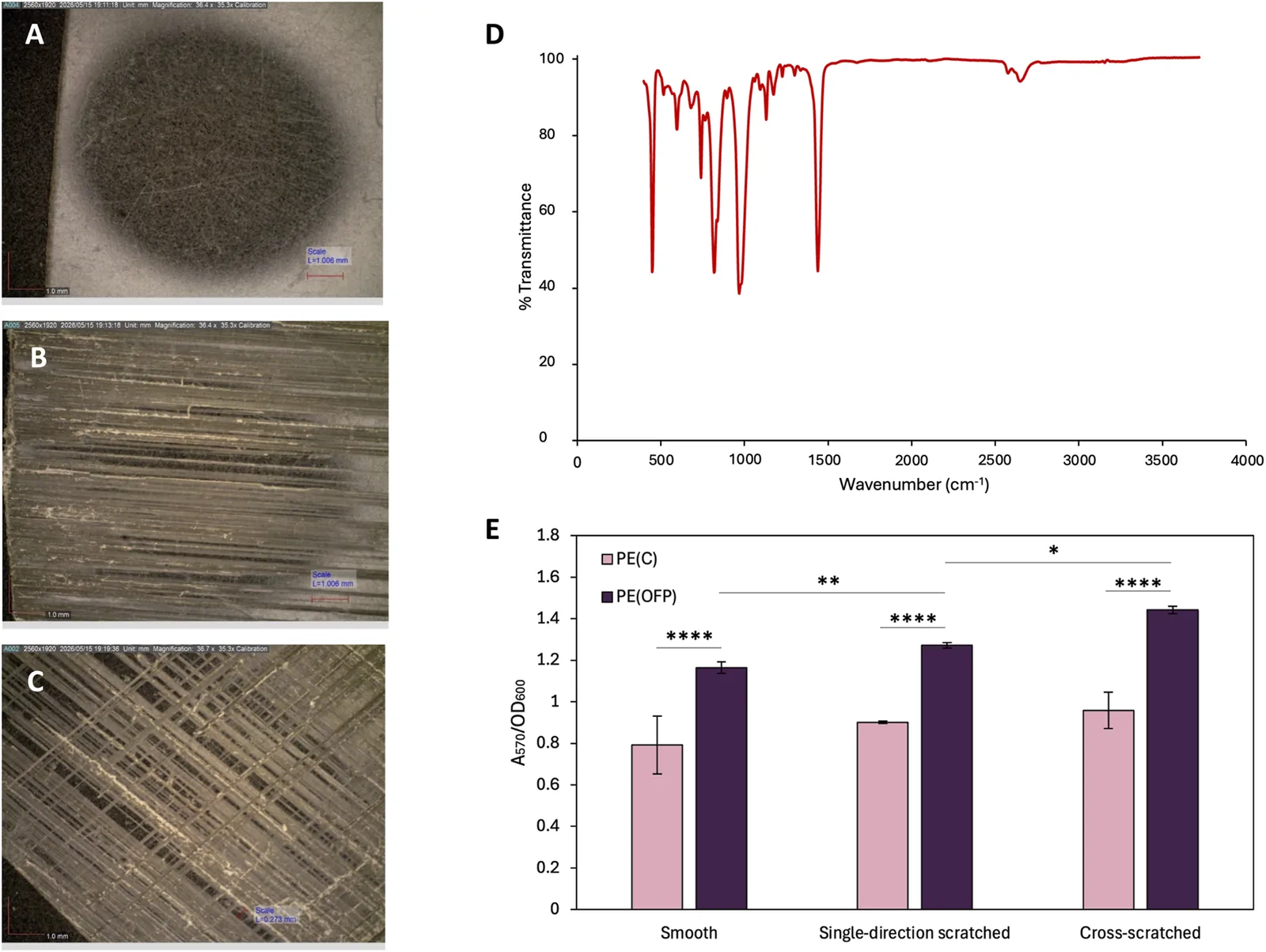
Effect of PET surface roughening on surface topography and biofilm formation. Representative optical microscopy images of PET films with **(A)** smooth, **(B)** single-direction scratched, and **(C)** cross-scratched surfaces. **(D)** Micro ATR-FTIR spectrum of roughened PET. **(E)** Biofilm formed by PE(C) and PE(OFP) on PET films with smooth, single-direction scratched, and cross-scratched surfaces. Biofilm formation was quantified by crystal violet staining and normalized to cell density (A_570_/OD_600_). Bars represent mean ± SD. Statistical significance is indicated by asterisks (*, p < 0.05; **, p < 0.01; ****, p < 0.0001).

Single-direction scratching produced the largest local peaks and valleys, with higher maximum peak height (Pp), maximum valley depth (Pv), peak-to-valley height (Pz/Pt), and root-mean-square height deviation (Pq) than cross-scratched films. Specifically, single-direction scratched films had Pp, Pv, Pz/Pt, and Pq values of 25.5 ± 16.5 µm, 27.0 ± 12.3 µm, 52.5 ± 26.9 µm, and 7.60 ± 3.99 µm, respectively, compared with 13.4 ± 2.6 µm, 15.3 ± 5.2 µm, 28.7 ± 5.9 µm, and 5.35 ± 2.26 µm for cross-scratched films (Supplementary Table S4). However, representative primary profiles show that these larger height deviations were more localized in the single-direction scratched films, with several pronounced peaks and valleys separated by relatively flatter regions (Supplementary Figure S2B). In contrast, cross-scratched films showed height variation distributed more continuously across the scan length (Supplementary Figure S2C). Together with the optical microscopy images, these results suggest that cross-scratching did not increase the maximum roughness amplitude, but generated more spatially distributed surface features that may provide more consistent attachment opportunities across the PET surface. Micro ATR-FTIR analysis showed no substantial differences between roughened (Figure 5D) and untreated PET films (Supplementary Figure S3), indicating that the treatment primarily modified surface topography rather than causing detectable changes in PET surface chemistry.

### 3.5 Biofilm formation on PET films

Since PE(C) and PE(OFP) showed the weakest and strongest PET surface attachment, respectively, these two conditions were selected for quantitative biofilm analysis on smooth, single-direction scratched, and cross-scratched PET films (Figure 5E). Biofilm quantification revealed that PE(OFP) consistently formed more biofilm than the control co-culture PE(C), showing an approximately 1.5-fold increase across the tested surfaces. Two-way ANOVA confirmed a significant effect of consortium composition on biofilm formation (F_1,12_ = 275.57, p = 1.21 × 10^−9^, Supplementary Table S5). This result indicates that extracellular PETase expression combined with the co-culture partner corresponds to higher surface-associated biomass than the baseline observed in the control consortium.

Surface roughening also significantly increased biofilm formation (F_2,12_ = 25.32, p = 4.94 × 10^−5^), with no significant interaction between surface treatment and consortium composition (F_2,12_ = 2.82, p = 0.099). Relative to smooth PET films, single-direction scratching increased biofilm formation by approximately 10%, while cross-scratched films produced an increase of approximately 24%. This trend is consistent with the surface characterization results, as cross-scratched films contained more frequent surface features than single-direction scratched films. The highest level of biofilm formation was observed for PE(OFP) on cross-scratched PET films, suggesting that the engineered expression construct, co-culture context, and increased surface feature density together favor microbial attachment.

## 4 Discussion

This study demonstrates that early microbial colonization of PET films is shaped by extracellular enzyme targeting, co-culture design, and surface topography. Most PET bioprocessing strategies focus on improving hydrolase activity, thermostability, or polymer accessibility; however, whole-cell systems also require cells and enzymes to interact effectively with an insoluble polymer interface. Our findings highlight surface attachment and biofilm formation as additional factors that strongly influence how effectively microbial systems engage with the polymer surface.

A key result of this work is the difference in extracellular FASTPETase recovery between E(FP) and E(OFP). SDS–PAGE analysis, combined with His-tag enrichment and Bradford-based protein estimation, showed detectable recovery of FASTPETase from the extracellular fraction of E(OFP), whereas no comparable recovery was observed from the matched E(FP) control. Because E(FP) and E(OFP) were cultivated and processed under identical conditions, recovery of FASTPETase only from E(OFP) is consistent with OmpA-dependent extracellular accumulation rather than with nonspecific culture-wide lysis. Although signal peptides such as OmpA and PelB are commonly used to direct recombinant proteins to the periplasm through the Sec-dependent pathway in *E. coli* (Karyolaimos et al., 2019; Crane and Randall, 2017), extracellular accumulation of signal peptide-fused proteins has also been reported in several cases. This may occur through periplasmic leakage, changes in outer-membrane permeability, or vesicle-associated release, depending on the signal peptide, target protein, host strain, and cultivation conditions (Mergulhão et al., 2005; Rinas and Hoffmann, 2004; Baumgarten et al., 2018). Several Sec-dependent signal peptides, including MalE, LamB, and PelB, have been used to obtain extracellular PETase from recombinant *E. coli* (Seo et al., 2019; Shi et al., 2021). For example, PelB-mediated targeting has been shown to promote extracellular accumulation of PETase in *E. coli* culture supernatants (Shi et al., 2021). In addition, OmpA has been used to facilitate extracellular production of recombinant enzymes such as *Bacillus subtilis* chitosanase and *Streptomyces* PMF phospholipase D in *E. coli*, indicating that OmpA-tagged proteins can, in some cases, accumulate in the culture supernatant (Pechsrichuang et al., 2016; Wang et al., 2020). Because PET hydrolysis occurs at the polymer surface, extracellular protein availability is important for improving enzyme access to the substrate. This result supports extracellular PETase availability as a contributor to the stronger PET surface attachment later observed in PE(OFP). Although OmpA fusion enabled detectable extracellular recovery of FASTPETase, extracellular enzyme availability may still be improved. The observed periplasmic enrichment identifies the OmpA targeting module as a promising target for further optimization. Engineering the OmpA signal sequence, optimizing its junction with FASTPETase, or incorporating signal-peptide enhancers can increase extracellular enzyme recovery, as previously demonstrated for engineered OmpA-based targeting of IsPETase in *E. coli* (Cui et al., 2021).

Comparison of the PET-film experiments further showed that co-culture composition strongly affected microbial attachment. Minimal attachment was observed in the control conditions C0 and PE(C), whereas PETase-expressing strains displayed clear differences in their ability to interact with the PET surface. E(OFP) showed detectable attachment even in monoculture, indicating that extracellular PETase availability was associated with early interaction with the polymer. However, the most extensive and persistent surface-associated biomass was observed in PE(OFP), suggesting that the combination of extracellular PETase secretion and the presence of *P. putida* provides a more favorable system for stable attachment and biofilm formation on PET.

The contrast between PE(FP) and PE(OFP) is particularly informative. Although both conditions involved co-culture with *P. putida*, the condition lacking the OmpA signal peptide showed weaker and less stable attachment over time. This suggests that the presence of the co-culture partner alone is not sufficient to maximize PET surface interaction, and that improved extracellular availability of PETase is also associated with more sustained attachment at the polymer interface. In addition, 16S rRNA analysis revealed a higher relative abundance of *E. coli* in PE(OFP) than in PE(FP), indicating that the OmpA-FASTPETase condition was associated with a difference in endpoint consortium composition. This observation may reflect differences in interactions between the consortium members and the PET surface, although further analysis would be needed to establish the underlying mechanisms. Together, these observations support the idea that enzyme access and community-level interactions must work together to strengthen microbial association with PET.

SEM imaging was consistent with the light microscopy results and confirmed the differences in PET surface attachment among the tested conditions. PE(C) showed little to no detectable surface-associated cells, whereas E(OFP), PE(FP), and especially PE(OFP) showed progressively greater coverage of the PET surface. In PE(OFP), dense cell clusters were observed, consistent with stronger surface-associated growth and biofilm development. This enhanced surface colonization was consistent with the higher relative abundance of *E. coli* in PE(OFP), suggesting that the OmpA–FASTPETase construct may be associated with both stronger PET attachment and differences in consortium composition. However, despite the clear differences in cell accumulation, no visible signs of PET erosion or pitting were detected within the experimental timeframe.

The importance of microbial attachment and biofilm formation on PET has also been demonstrated in studies of the naturally occurring marine isolates *R. pyridinivorans* P23 and *P. chengduensis* BC1815, both of which degrade PET using membrane-anchored esterases (Guo et al., 2023; Guo et al., 2025). In P23, biofilm formation was proposed to facilitate enzyme–substrate interactions and retain degradation intermediates near the polymer surface, while BC1815 further integrates PET degradation with ethylene glycol utilization. These observations highlight the functional relevance of surface colonization and monomer assimilation in PET-associated microbial systems. This study examines related interfacial factors through an engineered consortium that combines extracellular FASTPETase targeting in *E. coli* with a biofilm-forming, ethylene glycol-adapted *P. putida* partner, while further examining how PET surface topography influences biofilm development.

The roughening experiments further emphasized the importance of the physical properties of the substrate. Surface characterization indicated that the mechanical treatment primarily altered PET topography rather than producing detectable changes in surface chemistry, as roughened and untreated PET films showed comparable micro ATR-FTIR spectra with characteristic PET features (Kauts et al., 2023). Although previous studies have reported changes in PET surface chemistry following prolonged abrasion with sand or sand–gravel mixtures, these effects were observed over substantially longer exposure periods (Ziemann et al., 2025; Galea et al., 2025; Panczyk et al., 2024). Biofilm quantification showed that PE(OFP) produced more biofilm than PE(C) across all surface conditions, and that both single-direction scratched and cross-scratched films supported greater biomass than smooth PET films. The highest biofilm level was observed for PE(OFP) on cross-scratched PET. These results indicate that physical surface features can substantially affect attachment, likely by improving cell retention and providing a more favorable surface for early biofilm development.

The surface roughness analysis provides a physical explanation for the biofilm trends observed on roughened PET films. Mechanical abrasion increased surface height variation without substantially altering PET chemistry, suggesting that the enhanced biofilm formation was primarily driven by topographical changes. Importantly, the two abrasion patterns modified the surface in different ways. Single-direction scratching produced more dramatic local peaks and valleys, whereas cross-scratching generated a greater number of more closely spaced and more consistently distributed surface features. This greater feature density likely increased the number of potential attachment sites and improved early microbial retention at the PET interface, even though the individual height deviations were less pronounced than those produced by single-direction abrasion. These findings suggest that PET surface accessibility is governed not only by the magnitude of roughness, but also by the spacing, density, and distribution of surface features.

The present study examines early PET-surface colonization rather than PET depolymerization. The strongest effects were observed in microbial attachment and biofilm formation, supporting the importance of extracellular enzyme targeting, consortium composition, and surface accessibility at the polymer interface. The EG-utilizing consortium used here provides a metabolic basis for future systems in which PET-surface colonization is integrated with PET hydrolysis and downstream assimilation of the released monomers. Within the experimental timeframe, the primary observed outcomes were enhanced attachment and biofilm formation, while SEM revealed no visible surface pitting or erosion. Future work combining extracellular FASTPETase activity assessment with quantitative hydrolysis and product-release analyses will determine how microbial colonization influences polymer breakdown.

These results indicate that effective PET surface engagement depends not only on enzyme expression, but also on extracellular enzyme localization, microbial community organization, and polymer surface topography. By identifying factors that improve early attachment and biofilm formation, this work provides a foundation for future engineering of whole-cell PET bioprocessing systems. Building on this framework, future studies combining direct measurements of extracellular FASTPETase activity with quantitative analysis of PET hydrolysis products will establish the functional activity of the recovered enzyme and determine how PET-surface colonization influences PET hydrolysis.

## 5 Conclusion

This study presents a synthetic bacterial consortium designed to improve microbial colonization of PET surfaces through the combination of extracellular FASTPETase targeting, biofilm-promoting co-culture design, and PET surface roughening. The experiments demonstrate differences in bacterial attachment and biofilm formation; however, no visible surface pitting or erosion was observed by SEM analysis within the experimental timeframe. These findings identify extracellular enzyme targeting, consortium composition, and surface topography as factors affecting early PET colonization and provide a foundation for future studies coupling improved colonization with quantitative PET hydrolysis and downstream monomer utilization.

## Data Availability

The original contributions presented in the study are included in the article/Supplementary Material, further inquiries can be directed to the corresponding author.
