# Supplementary material for: Engineering PET surface colonization with a synthetic bacterial consortium
Source: Front Bioeng Biotechnol. 2026 Sep 30;14:1951515. doi: 10.3389/fbioe.2026.1951515 (PMC13662134; doi:10.3389/fbioe.2026.1951515)
Supplement: Supplementary file 1 [file DataSheet1.pdf]

Supplementary Materials

Engineering PET Surface Colonization with a Synthetic Bacterial Consortium

Bahareh Arab, Joseph Wortman, Boxin Zhao, C. Perry Chou, Yilan Liu

Department of Chemical Engineering, University of Waterloo, Waterloo, Ontario, N2L 3G1, CA

* Corresponding author:
Yilan Liu
Department of Chemical Engineering
University of Waterloo
200 University Avenue West
Waterloo, Ontario, Canada N2L 3G1
E-mail: yilan.liu@uwaterloo.ca
Telephone: 1-519-888-4567 ext. 40641

Table S1: Strains and plasmids used in this study

Name	Description or relevant genotype	Source
**Host strains**		
HI-Control 10G	*mcrA,* Δ(*mrr-hsdRMS-mcrBC*), *endA1, recA1, ϕ*80*dlacZ*ΔM15, Δ*lacX74, araD139,* Δ(*ara leu*)*7697, galU, galK, rpsL* (Str^R), *nupG, λ⁻* , *tonA,* Mini-F *lacI*^{q1} ($Gent^R$)	Lucigen
EcNev	*Escherichia coli* Nissle 1917, evolved on ethylene glycol	[1]
Ppev	*Pseudomonas putida* KT2440, evolved on 1,6-hexamethylenediamine and ethylene glycol	[1, 2]
PE(C)	Ppev + EcNev/pTrc99a	This study
E(OFP)	EcNev/OFP	This study
E(FP)	EcNev/FP	This study
PE(FP)	Ppev + EcNev/FP	This study
PE(OFP)	Ppev + EcNev/OFP	This study
**Plasmids**		
pTrc99a	pBR322 ori, Amp^R	Lab stock
OFP	pBR322 ori, Amp^R, P_{J23119}::(OmpA:*FASTPETase*:His_6)	This study
FP	pBR322 ori, Amp^R, P_{J23119}::*FASTPETase*:His_6	This study

Table S2: Roughness profile peak amplitude parameters of the control sample scans.

Data Point and Abrasion Dimensions	Roughness Profile Peak Amplitude Parameters								
	Rp (µm)	Rv (µm)	Rz (µm)	Rc (µm)	Rt (µm)	Ra (µm)	Rq (µm)	Rsk (-)	Rku (-)
Control 1	0.05	0.02	0.07	0.02	0.07	0.01	0.01	1.80	9.96
Control 2	0.08	0.03	0.11	0.03	0.11	0.01	0.01	1.28	9.33
Control 3	0.05	0.02	0.07	0.02	0.07	0.01	0.01	0.99	6.76
Control 4	0.21	0.05	0.26	0.04	0.26	0.01	0.02	6.97	82.39
Average ± SD	0.10 ± 0.08	0.03 ± 0.01	0.13 ± 0.09	0.03 ± 0.01	0.13 ± 0.09	0.01 ± 0.00	0.01 ± 0.00	2.76 ± 2.83	27.11 ± 36.88

Table S3: Roughness profile peak density of untreated PET control films

Data Point and Abrasion Dimensions	Roughness Profile Peak Density
	RPc (+/-0.5 µm) (mm^{-1})
Control 1	0
Control 2	0
Control 3	0
Control 4	0

Table S4: Primary profile peak amplitude parameters of both the one-dimensional and two-dimensional abrasion.

Data Point and Abrasion Dimensions	Primary Profile Peak Amplitude Parameters								
	Pp (µm)	Pv (µm)	Pz (µm)	Pc (µm)	Pt (µm)	Pa (µm)	Pq (µm)	Psk (-)	Pku (-)
Single-direction 1	32.70	19.18	51.88	25.50	51.88	4.33	7.96	1.91	9.58
Single-direction 2	45.03	45.16	90.19	31.04	90.19	8.45	12.87	-0.14	6.00
Single-direction 3	15.45	24.11	39.57	18.86	39.57	4.12	6.16	-1.39	6.60
Single-direction 4	8.67	19.70	28.37	12.52	28.37	1.78	3.40	-2.65	14.04
Cross-scratched 1	10.90	16.26	27.17	13.81	27.17	5.01	6.35	-0.91	2.94
Cross-scratched 2	14.94	22.22	37.16	17.88	37.16	5.94	8.04	-0.87	3.79
Cross-scratched 3	11.51	11.80	23.31	10.65	23.31	2.60	3.90	0.05	4.66
Cross-scratched 4	16.15	10.97	27.12	8.51	27.12	1.66	3.11	1.41	11.69
Single-direction (Average ± SD)	25.46 ± 16.51	27.04 ± 12.28	52.50 ± 26.90	21.98 ± 8.04	52.50 ± 26.90	4.67 ± 2.77	7.60 ± 3.99	-0.57 ± 1.94	9.06 ± 3.67
Cross-scratched (Average ± SD)	13.38 ± 2.56	15.31 ± 5.16	28.69 ± 5.93	12.71 ± 4.08	28.69 ± 5.93	3.80 ± 2.00	5.355 ± 2.26	-0.08 ± 1.09	5.77 ±4.01

Table S5: Two-way ANOVA of the effects of consortium composition and PET surface treatment on biofilm formation.

SUMMARY	PE(C)	PE(OFP)	Total
Smooth			
Count	3	3	6
Sum	2.31396	3.47707	5.79103
Average	0.77132	1.15902	0.96517
Variance	0.01101	0.00044	0.04968
Single-direction scratched			
Count	3	3	6
Sum	2.69135	3.82472	6.51608
Average	0.89712	1.27491	1.08601
Variance	0.00005	0.00011	0.04288
Cross-scratched			
Count	3	3	6
Sum	2.79794	4.33265	7.13059
Average	0.93265	1.44422	1.18843
Variance	0.00595	0.00019	0.08097
Total			
Count	9	9	
Sum	7.80325	11.63445	
Average	0.86703	1.29272	
Variance	0.00964	0.01561	

ANOVA

Source of Variation	*SS*	*df*	*MS*	*F*	*P-value*	*F crit*
Sample	0.14988	2	0.07494	25.32387	4.93918E-05	3.88529
Columns	0.81545	1	0.81545	275.56537	1.21309E-09	4.74723
Interaction	0.01667	2	0.00833	2.81654	0.099338994	3.88529
Within	0.03551	12	0.00296			
Total	1.01750	17				

Comparison	Mean difference	Adjusted (p)-value	Significance
Single-scratched vs. smooth	+0.1208	0.0061	**
Cross-scratched vs. single-scratched	+0.1024	0.0174	*

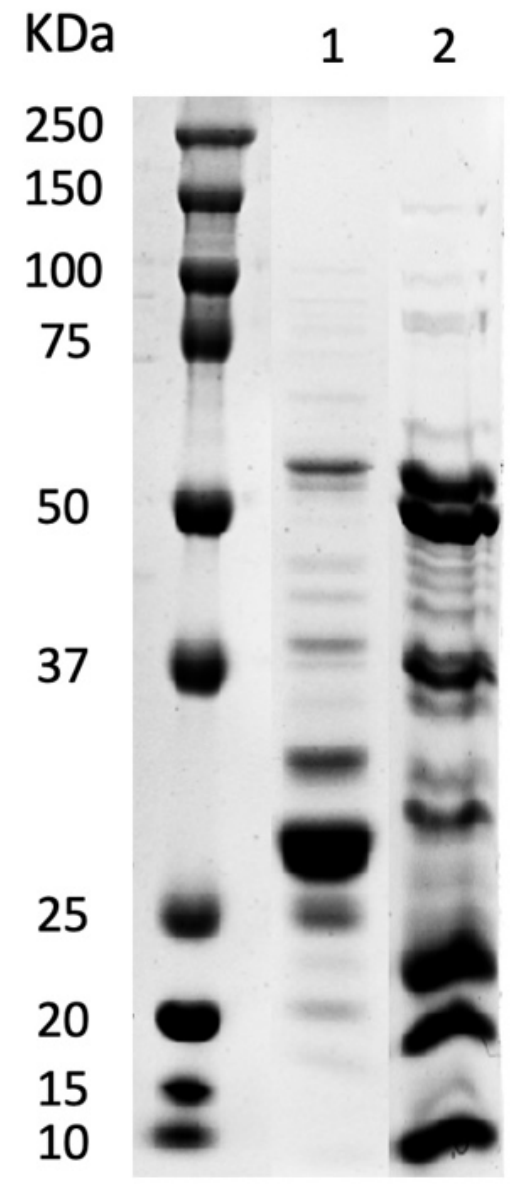

Figure S1. Subcellular localization of OmpA-FASTPETase in E(OFP). SDS–PAGE analysis of the periplasmic fraction (Lane 1) and remaining cellular lysate collected after periplasmic extraction (Lane 2)

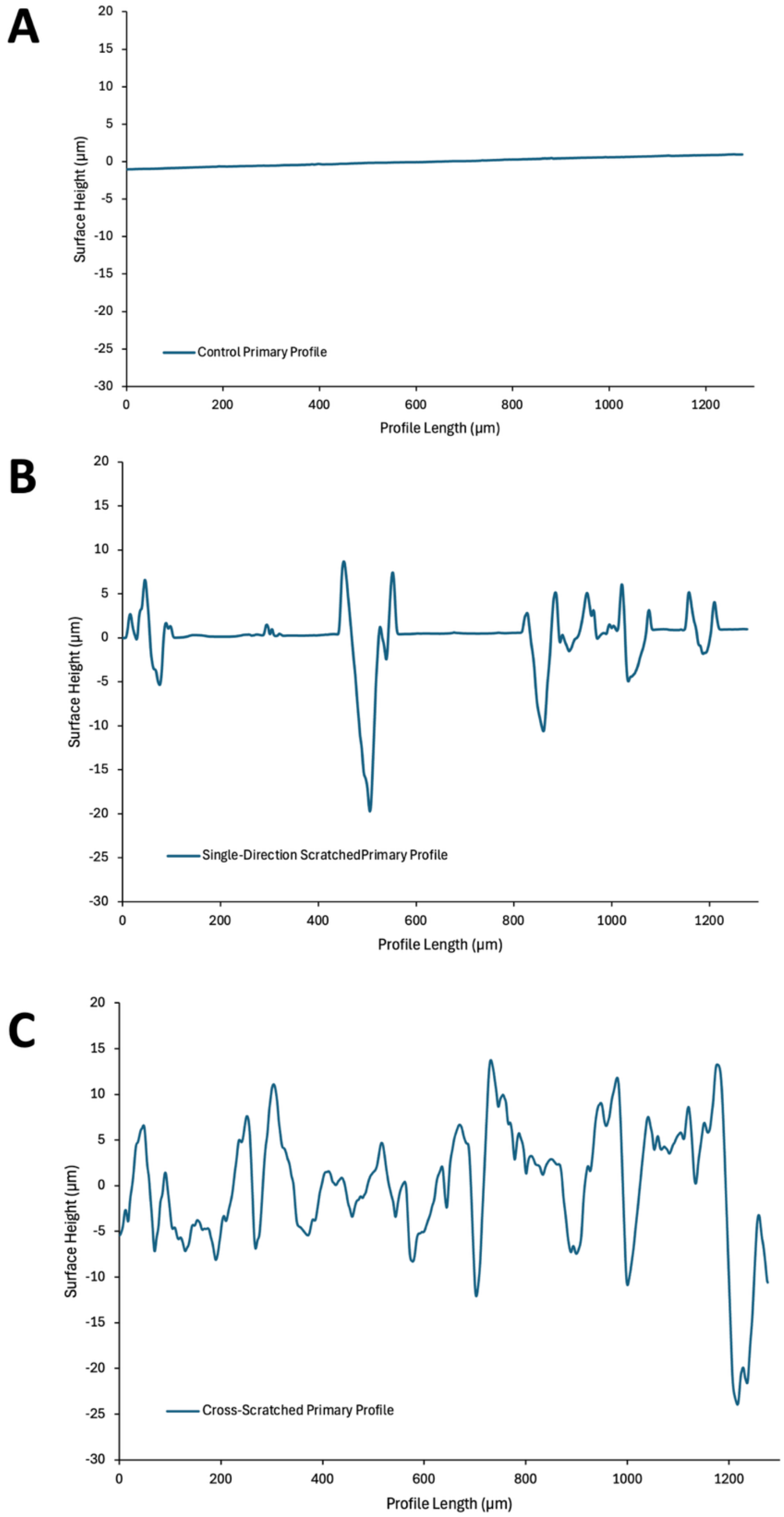

Fig. S2: Representative profilometer profiles of untreated and roughened PET films. Primary profiles of (A) untreated PET showing slight sample warpage; a Gaussian cut-off filter was applied before calculating the roughness parameters reported in Tables S2 and S3. Primary profiles of (B) single-direction scratched and (C) cross-scratched PET films.

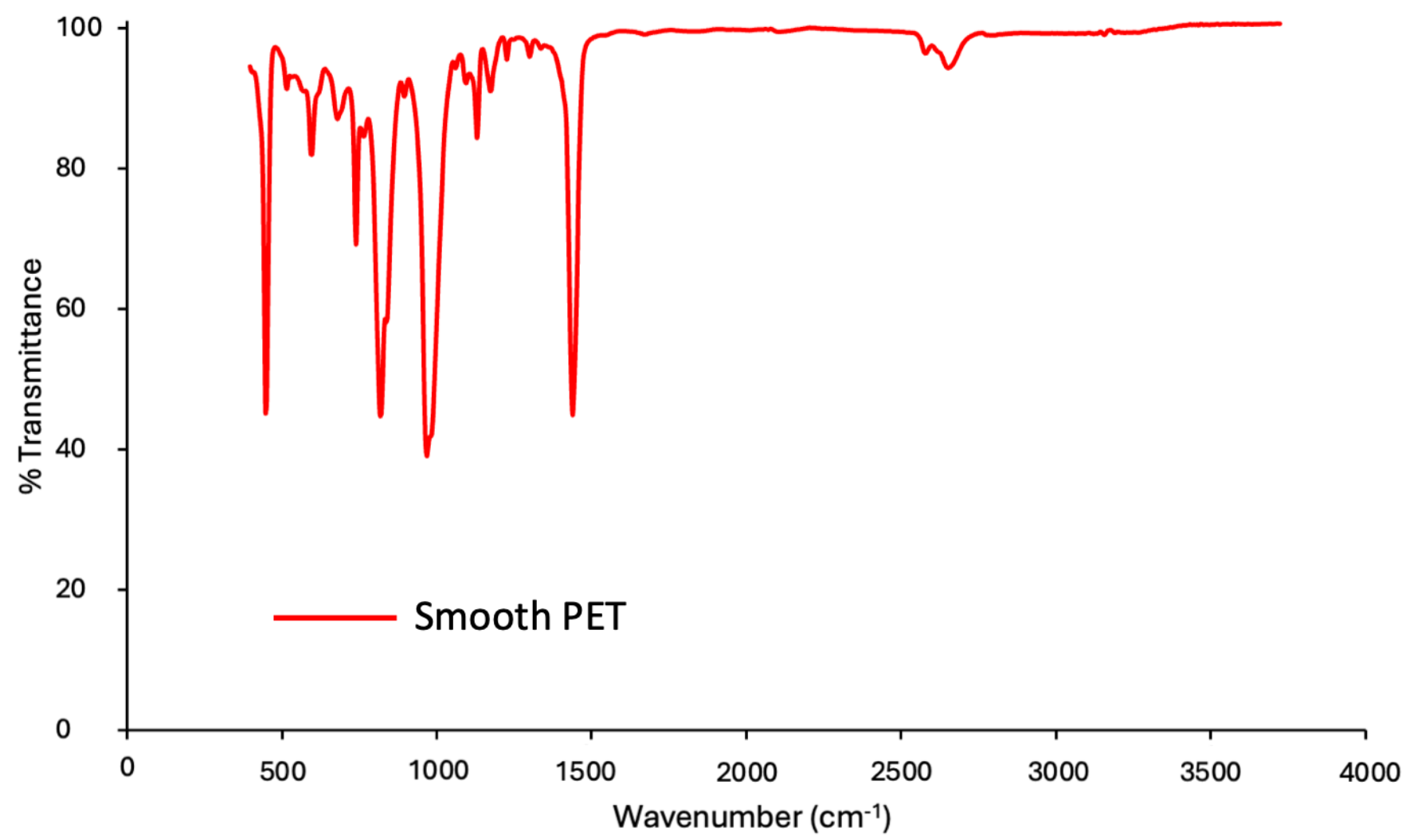

Fig. S3: Micro ATR-FTIR spectrum of smooth PET

References

1. Dharmasiddhi, I.P.W., et al., *Engineering a cross-feeding synthetic bacterial consortium for degrading mixed PET and nylon monomers.* Processes, 2025. **13**(2): p. 375.
2. Arab, B., et al., *Advancing bio-recycling of nylon monomers through CRISPR-assisted engineering.* Environmental Technology & Innovation, 2025. **39**: p. 104267.
